# A reverse-phase protein microarray-based screen identifies host signaling dynamics upon *Burkholderia* spp. infection

**DOI:** 10.3389/fmicb.2015.00683

**Published:** 2015-07-27

**Authors:** Chih-Yuan Chiang, Ijeoma Uzoma, Douglas J. Lane, Vesna Memišević, Farhang Alem, Kuan Yao, Krishna P. Kota, Sina Bavari, Anders Wallqvist, Ramin M. Hakami, Rekha G. Panchal

**Affiliations:** ^1^Molecular and Translational Sciences Division, United States Army Medical Research Institute of Infectious Diseases, FrederickMD, USA; ^2^Department of Defense Biotechnology High Performance Computing Software Applications Institute, Telemedicine and Advanced Technology Research Center, United States Army Medical Research and Materiel Command, FrederickMD, USA; ^3^National Center for Biodefense and Infectious Diseases, and School of Systems Biology, George Mason University, ManassasVA, USA; ^4^PerkinElmer, Inc., WalthamMA, USA

**Keywords:** *Burkholderia mallei*, *Burkholderia pseudomallei*, lipopolysaccharide, reverse-phase protein microarrays

## Abstract

*Burkholderia* is a diverse genus of gram-negative bacteria that causes high mortality rate in humans, equines and cattle. The lack of effective therapeutic treatments poses serious public health threats. Developing insights toward host-*Burkholderia* spp. interaction is critical for understanding the pathogenesis of infection as well as identifying therapeutic targets for drug development. Reverse-phase protein microarray technology was previously proven to identify and characterize novel biomarkers and molecular signatures associated with infectious disease and cancer. In the present study, this technology was utilized to interrogate changes in host protein expression and phosphorylation events in macrophages infected with a collection of geographically diverse strains of *Burkholderia* spp. The expression or phosphorylation state of 25 proteins was altered during *Burkholderia* spp. infections of which eight proteins were selected for further characterization by immunoblotting. Increased phosphorylation of AMPK-α1, Src, and GSK3β suggested the importance of their roles in regulating *Burkholderia* spp. mediated innate immune response. Modulating the inflammatory response by perturbing their activities may provide therapeutic routes for future treatments.

## Introduction

*Burkholderia pseudomallei* (*Bp*) and *Burkholderia mallei* (*Bm*) are facultative intracellular gram-negative bacterial pathogens that cause melioidosis and glanders, respectively. While genetically similar, *Bp* and *Bm* are associated with their own hallmarks. *Bp* is endemic to tropical regions of Southeast Asia, Northern Australia, and China, where it inhabits the soil and stagnant water. Routes of human infection include inhalation, ingestion, and contact with open wounds; however, human-to-human transmission is extremely rare (White, 2003; Gilad, 2007; Galyov et al., 2010). Symptomatic infection may present with flu-like symptoms such as fever and pulmonary distress, making accurate diagnosis of melioidosis difficult in the early stages (Dance, 1991; Leelarasamee, 2004; De Keulenaer and Cheng, 2006). Fatal infection is often due to progression to septicemia and acute pneumonia (Dance, 1991, 2000; Dharakul and Songsivilai, 1999; Galyov et al., 2010). By contrast, *Bm* is a non-motile obligate mammalian pathogen endemic among domestic animals in Africa, Asia, the Middle East, and Central and South America (Whitlock et al., 2007). Equine species are the natural reservoir for *Bm* and are responsible for transmission of glanders to humans through contact, leading to development of pneumonia and septicemia, often resulting in fatality (Whitlock et al., 2007; Galyov et al., 2010; Van Zandt et al., 2013). Given that both species are highly infectious via aerosolization, coupled with the lack of vaccines, *Bp* and *Bm* are considered category B bioterrorism agents by the U. S. Centers for Disease Control (Holden et al., 2004; Whitlock et al., 2007; Galyov et al., 2010).

*Bp* and *Bm* are resistant to many traditional antibiotics (Whitlock et al., 2007; Estes et al., 2010; Galyov et al., 2010). Although successful treatment is possible with several months of a combination antimicrobial regimen, relapse is common (Holden et al., 2004; Estes et al., 2010). Effort has been placed on evaluating the therapeutic potential of bacterial secretion systems and effectors as components of candidate vaccines (Whitlock et al., 2007); however, none are available at this time. Additional therapeutic strategies are required for controlling disease in endemic regions as well as for protection against potential bioterrorism threats. Immunotherapy in conjunction with antimicrobials is a new theme in the *Burkholderia* treatment paradigm. A promising study found that combining interferon (IFN)-γ with an antimicrobial showed synergistic inhibition of growth in *Bp* infected macrophages (Propst et al., 2010). Identification of additional host targets that function in resolving the overactive immune and inflammatory responses elicited by infection is an active area of research (Ulett et al., 2000).

Pattern recognition receptors (PRRs) are innate immune sensors that serve as the first line of defense against pathogen infections. Toll like receptor 4 (TLR4) is a PRR that detects lipopolysaccharide (LPS) expressed on the surface of *Burkholderia* spp. TLR4 activation recruits two adaptor proteins, myeloid differentiation primary response gene 88 (MyD88) and TIR-domain-containing adapter-inducing interferon-β (TRIF). The TRIF-dependent pathway mediates the upregulation of type I IFN and subsequent phosphorylation of Signal Transducers and Activators of Transcriptions (STATs). The MyD88-dependent pathway leads to the activation of IKKα and IKKβ, which specifically phosphorylate two serines on the cytoplasmic NF-κB inhibitor, IκB. Phosphorylation of IκB causes its ubiquitin-dependent degradation, leading to the activation and nuclear translocation of NF-κB (Akira et al., 2006). In addition to the NF-κB pathway, TLR4 also activates Stress Activated Protein Kinases (SAPK) such as p38 via apoptosis signal-regulating kinase 1 (ASK1), a MAP kinase kinase kinase (MEKK; Matsuzawa et al., 2005). Although surrogate ligand-mediated (e.g., LPS) pro-inflammatory pathway is extensively studied, the role of *Bp* and *Bm* LPS in human melioidosis or glanders is just beginning to unravel (Chantratita et al., 2013; Alam et al., 2014). Furthermore, larger molecular signaling networks that are critical for invasion, survival and replication of *Burkholderia* spp. in the host require characterization.

Reverse-phase protein microarray (RPMA) is a quantitative, high throughput tool that enables interrogation of changes in protein expression and post-translational modification in complex cellular signaling networks upon perturbation. This technology has been successfully utilized to profile molecular signatures and functional pathway activation associated with ovarian and breast cancer, respectively, (Sheehan et al., 2005; Wulfkuhle et al., 2012). Furthermore, phosphorylation states of key host signaling proteins were characterized in human samples infected with highly pathogenic agents such as Rift valley fever virus and *Yersinia pestis* (Popova et al., 2010; Alem et al., 2015). The goal of this study was to comprehensively examine whether diverse strains of *Bp, Bm*, and *Burkholderia thailandensis* (*Bt*) trigger signaling through common or distinct host pathways. To this end, we utilized a RPMA platform to simultaneously detect changes in the expression of host signaling proteins as well as alterations in phosphorylation-mediated host signaling caused by infection with *Burkholderia* spp. To generate host protein expression and phosphorylation profiles, RAW264.7 murine macrophages were infected with geographically diverse isolates of *Bp* and *Bm*, and at various time points protein lysates were examined using RPMA. This approach identified 25 candidates whose expression levels and/or phosphorylation states were altered, of which eight proteins were selected for further characterization through immunoblot analysis. Consistent with published studies, we identified phosphorylation of glycogen synthase kinase 3 beta (GSK3β) and components of the NF-κB and MAPK pathways, validating the RPMA approach. Moreover, we report phosphorylation of additional host proteins that were not previously linked to *Burkholderia* spp. infection, including AMP activated protein kinase (AMPK-α1) and Src. The signaling architecture is similar between *Burkholderia* spp. with minor distinctions in the magnitude of activation or induction for iNOS, phosphorylated GSK3β and STAT1 and c-Myc. Pharmacological perturbation of these critical cascades may serve as therapeutic routes for *Burkholderia* spp. infections.

## Materials and Methods

### Bacterial Strains

*Bm* ATCC 23344, NCTC 10247, NCTC 10229, NCTC 3708, NCTC 3709, 2002721278 (Burtnick et al., 2002); *Bp* E8, 576, MSHR305 (Bast et al., 2011; Van Zandt et al., 2012) and *Bt* DW503 were used in this study. *Burkholderia* spp. were maintained on Luria Broth (LB) plates with 1.5% agar or on 5% sheep blood agar (SBA) plates. *Bp* and *Bt* strains were cultured in LB whereas *Bm* strains were propagated in LB with 4% glycerol. Bacterial concentrations were quantified using optical density (OD) at 600 nm readings and diluted using a conversion factor of 5 × 10^8^ CFU/ml per unit of OD. All studies using viable *Bp* and *Bm* isolates were performed using biosafety level three conditions.

### Cell Lines

Mouse macrophage cell line RAW264.7 was obtained from ATCC (Manassas, VA). Cells were cultured in DMEM (Life Technologies) supplemented with 10% fetal bovine serum (Hyclone), 1% nonessential amino acids (Sigma-Aldrich), and 1% glutamax (Life Technologies), grown at 37°C in 5% CO_2_.

### Antibodies

For the RPMA studies, only validated antibodies known to cross react with murine target proteins were selected (**Supplementary Table S1**). For immunoblotting, the following antibodies were purchased from Cell Signaling Technology: iNOS (#2977), IκBα (#9242), phospho STAT1 (#9171), phospho p38 (#9211), phospho ERK1/2 (#9101), phospho AMPK-α1 (#4184), total p38 (#2387), phospho Src (#6943), c-Myc (#9402), phospho GSK3β (#9331). GAPDH was purchased from Sigma-Alrich (G9545). Total GSK3α/β was purchased from Santa Cruz biotechnology (SC-56913). Total ERK1 was purchased from BD Transduction laboratories (M12320).

### Infection of RAW264.7 Macrophages

RAW264.7 macrophages (1 × 10^6^ cells/well) were seeded overnight in six well plates. The next day, cells were infected with one *Bt*, five *Bm*, and three *Bp* strains at multiplicity of infection (MOI) of 1 and 10. In parallel, cells were stimulated with *Escherichia coli* LPS at 100 ng/ml. Uninfected and unstimulated cells were used as negative controls. RAW264.7 cell samples were collected at 30 min, 1, 4, and 8 h post *Burkholderia* spp. infection, washed with phosphate buffered saline (PBS) and lysed. For the longer incubation time points of 4 and 8 h, extracellular bacteria were removed 2 h post infection by washing the cells three times with PBS and then further incubated with pre-warmed DMEM containing 10% fetal bovine serum and 250 μg/ml of kanamycin (Km) (Brett et al., 2008) to reduce extracellular bacterial growth. All experiments were performed on two independent days and each biological sample lysate was printed in triplicate on nitrocellulose coated glass slides.

### Reverse Phase Antibody Array

For RPMA assays, cells were harvested, washed with PBS, and then lysed in a mixture of T-PER Reagent (Thermo Scientific) and 2X Tris-Glycine SDS sample buffer (Life Technologies), complete protease inhibitor cocktail (Roche), Na_3_VO_4_, NaF, EDTA, and DTT. RPMAs were constructed and analyzed as previously described (Improta et al., 2011; Federici et al., 2013). Samples from duplicate sets of *Burkholderia* spp. infections were printed in triplicate spots on nitrocellulose coated glass slides (GRACE Bio-Labs, Inc.) using an Aushon 2470 microarrayer equipped with 185 μm pins (Aushon Biosystems), according to manufacturer’s instructions. A subset of the printed array slides was stained with Spyro Ruby Protein Blot Stain (Life Technologies) to estimate sample total protein concentration. Before staining, proteins array slides were treated with 1X ReBlot Mild Solution (Chemicon) for 15 min, washed two times for 5 min in PBS (Life Technologies), and incubated for 1 h in blocking solution (2% I-Block, 0.1% Tween-20 in PBS; Life Technologies). Immunostaining was performed on an automated slide stainer using a signal amplification kit (DAKO). The arrays were probed with a collection of 114 antibodies. Primary antibody binding was detected using a biotinylated goat-anti rabbit immunoglobulin G (IgG) H + L at 1:7,500 dilution (Vector Laboratories) or rabbit anti-mouse IgG at 1:10 dilution (DAKO) followed by streptavidin-conjugated IRDye680 fluorophore (LI-COR Biosciences). All slides were scanned using a Revolution 4550 scanner (Vidar Systems Corporation), and acquired images were analyzed with MicroVigene v.4.0.0.0 (VigeneTech Inc.), which conducted spot detection, local background subtraction, negative control subtraction, replicate averaging, and total protein normalization, producing a single value for each sample.

### RPMA Data Analysis

Reverse-phase protein microarray data for 15 out of the 114 antibodies used in the study had expression values around zero. As we could not assess whether these values represent true biological values or are a consequence of experimental and array processing procedures, data from these 15 antibodies was not included in further analyses. Technical replicates were independently averaged to generate a value for each biological sample. Expression values for individual biological replicates were corrected by normalizing to the value of the uninfected control condition. Fold-change values were generated for each time point. Biological replicates were averaged and those for which the average fold change remained twofold or larger (i.e., fold-change ≤ 0.5 for under-expressed proteins or fold change ≥2 for over-expressed proteins) were included in further analysis.

### Immunoblotting

For immunoblot analysis, RAW264.7 macrophages infected at MOI 10 were pelleted and lysed in radioimmunoprecipitation assay (RIPA) buffer (Thermo Scientific) containing complete protease inhibitor cocktail (Roche), and phosphatase inhibitors (Roche). The amount of total protein in each sample was quantified by Bradford assay (Bio-Rad). RAW264.7 cell lysate preparations were boiled for 10 min with NuPAGE LDS Sample Buffer (Life Technologies) containing 10% 2-Mercaptoethanol. Equivalent amounts of total protein were loaded onto 4–12% Bis-Tris gradient gels (Life Technologies) and electrophoresed. Following electrophoresis, proteins were transferred to a nitrocellulose membrane (Life Technologies) and then blocked in 5% bovine serum albumin for 1 h prior to incubation with primary antibodies overnight. Blots were washed, then incubated with appropriate HRP-conjugated secondary antibodies (Jackson ImmunoResearch). Signal was detected by ECL Prime (GE Healthcare Life Sciences). Image acquisition was performed with ChemiDoc^TM^ XRS+ system (Bio-Rad). Relative protein levels were normalized with endogenous GAPDH as a loading control.

### Immuno-Fluorescence Staining

RAW264.7 macrophages (4 × 10^4^ cells/well) were seeded in a Falcon 96 well flat bottom plate (REF353219). Cells were stimulated with 1 μg/ml of LPS or infected with *Bm* ATCC23344, *Bm* 2002721278, and *Bp* E8 at MOI of 10. At 30 min, 1, 4 and 8 h post infection, cells were washed and fixed with formaldehyde. Immuno-fluorescent staining of phospho ERK1/2 (Cell Signaling Technology #9101) was performed according to the manufacturer’s protocol. Images were acquired by Opera confocal reader (model 3842) using a 20X water objective. Analysis of the images was accomplished within the Opera system using standard Acapella scripts.

### Colony Forming Unit (CFU) Assay to Quantify Bacterial Uptake and Intracellular Replication

RAW264.7 macrophages (2 × 10^5^ cells/well) were seeded in 24 well tissue culture plates and incubated overnight at 37°C with 5% CO_2_. Cells were infected with *Burkholderia* spp. at an MOI of 10, and after 1 h infected cells were treated with or without aminoguandine (200 μg/ml, Sigma-Aldrich; Brett et al., 2008). Two hours post-infection, cells were washed three times with PBS to remove extracellular bacteria and then further incubated with pre-warmed DMEM containing 10% fetal bovine serum and 250 μg/ml of Km. At 3 and 24 h post-infection, supernatants were harvested and macrophage monolayers were washed two times with PBS and lysed with 0.1% (vol/vol) Triton X-100. Serial dilutions of the lysates were plated onto SBA plates. After incubation for 48 h at 37°C, colonies were counted and CFU/ml was computed.

### Quantification of Nitrite and Murine IFN-β

Supernatants harvested from the above assay were evaluated for nitrite and IFN-β production using a Nitric Oxide Assay Kit (Thermo Scientific) and mouse IFN-β ELISA kit (Thermo Scientific), respectively.

## Results

### Identification of Host Factors that were Differentially Expressed upon *Burkholderia* spp. Infection

**FIGURE 1:**
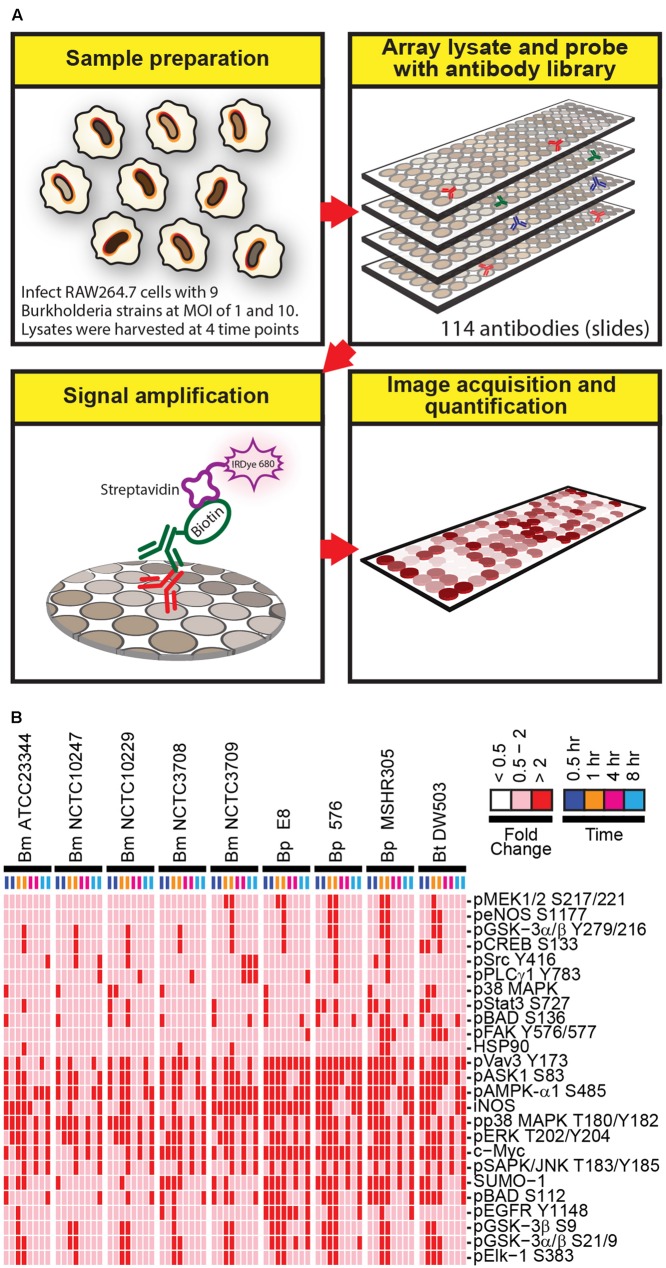
**A schematic diagram of the experimental design and overview of the results. (A)** Lysates harvested from *Burkholderia* spp. infected RAW264.7 macrophages were arrayed on nitrocellulose coated slides. Each slide was incubated with one primary antibody with a total of 114 antibodies in the library. The primary antibody was detected by biotin-labeled secondary antibody, which, in turn, was recognized by IRDye680 fluorophore conjugated streptavidin. Signals were acquired, quantified, and analyzed according to materials and methods section. **(B)** RAW264.7 macrophages were infected at multiplicity of infection (MOI) of 10 with indicated *Burkholderia* spp. for 0.5, 1, 4, and 8 h. Lysates were harvested and subjected to reverse-phase protein microarray (RPMA) studies. A heat map of fold changes over untreated samples is depicted. Experiments were performed on two independent days and data from these repeat studies are shown.

To gain insight into host proteins that are differentially expressed or post-translationally modified following *Burkholderia* spp. infection, a high throughput RPMA-based screening platform was utilized. RAW264.7 macrophage were adopted due to its ability to phagocytose *Burkholderia* spp., which in turn exploits the host’s cellular systems by promoting host actin polymerization on the bacterial surface and inducing multinucleated giant cells (MNGCs) formation (Galyov et al., 2010; Pegoraro et al., 2014). Host responses to a collection of nine *Burkholderia* spp. originating from various geographical locations isolated from human, animals, or environmental sources were investigated. The ancestry for the majority of the strains is known along with their virulence profiles and genome sequences. RAW264.7 macrophage lysates harvested at various time points post *Burkholderia* spp. infection were individually arrayed onto nitrocellulose coated slides and probed with a total of 114 well characterized antibodies (**Figure 1A**). Statistical analysis revealed 25 candidates whose change in expression was twofold or higher in both replicates in any strain, at any time point and at MOI of 10 (**Figure 1B**; **Supplementary Figure S1**). At MOI of 1, very few robust changes were observed in host signaling molecules. Hence, subsequent studies were performed using MOI of 10. RPMA data analysis did not identify any proteins whose expression was considerably down regulated compared to uninfected and untreated control. The selected candidates were further characterized by performing traditional immunoblots using cell lysates collected from RAW264.7 macrophage treated with LPS, or infected with *Bm* ATCC23344, and *Bp* E8. Although not included in the initial RPMA, *Bm* 2002721278, an avirulent strain (personal communication), was included for purposes of comparative analyses of host signaling dynamics between virulent and avirulent *Bm*.

### *Burkholderia* spp. Infection Induces iNOS Expression and Activation of STAT1

**FIGURE 2:**
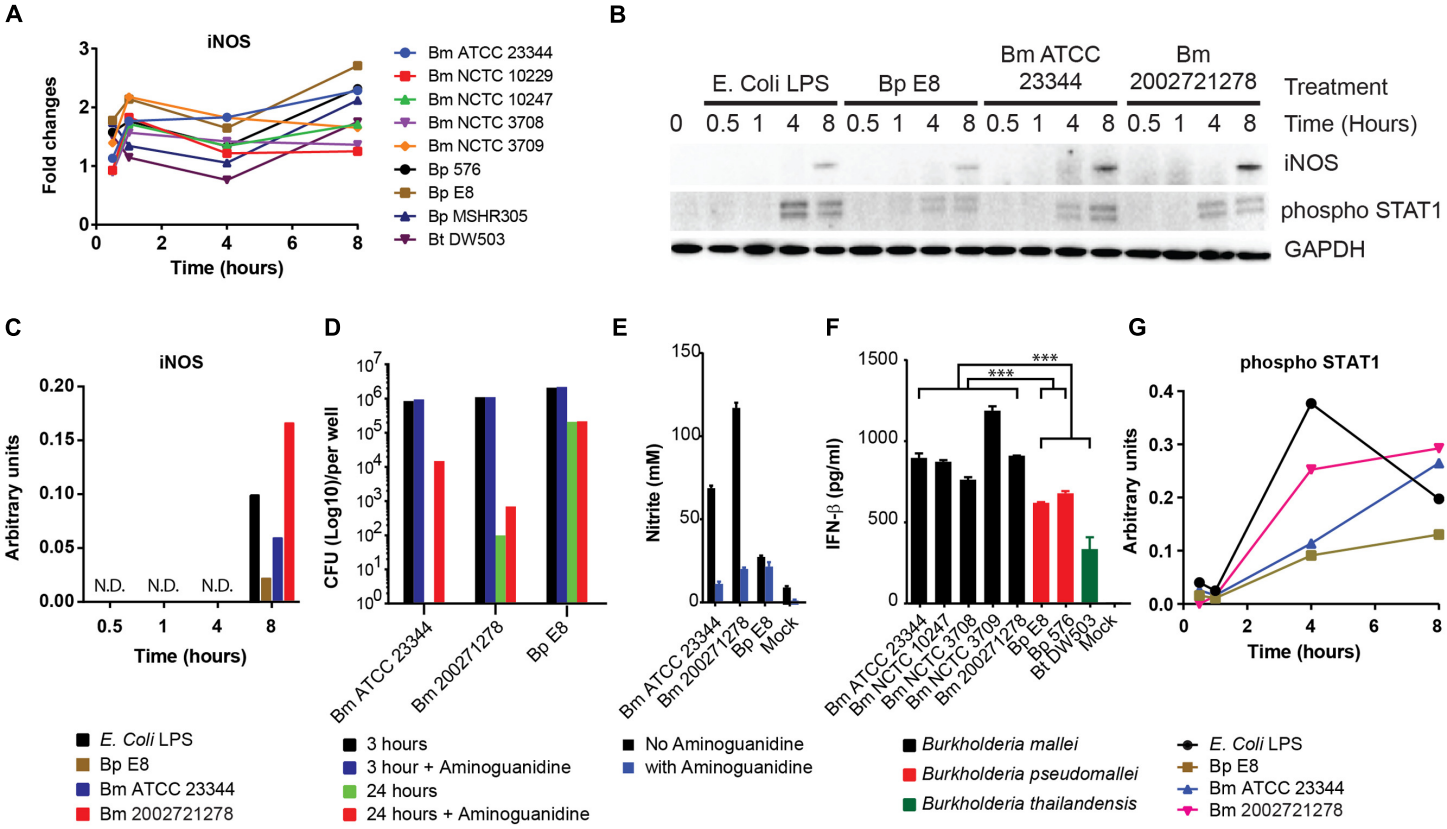
**Induction of iNOS and activation of STAT1 in response to *Burkholderia* spp. infection. A)** The expression pattern of iNOS obtained from RPMA study. **(B)** RAW264.7 macrophages were infected with *Burkholderia pseudomallei (Bp)* E8, *Burkholderia mallei (Bm)* 23344, and *Bm* 2002721278 at MOI of 10, or treated with LPS at 100 ng/ml. Samples were collected at 0.5, 1, 4, and 8 h post treatments. The amount of iNOS and phosphorylated STAT1 was evaluated by immunoblotting, followed by **(C)** densitometric quantification of iNOS. N.D. indicates signal was not detectable. **(D)** Colony forming unit (CFU) was determined 3 and 24 h post *Burkholderia* spp. infection of RAW264.7 cells, with and without aminoguanidine (AG) treatment. Culture supernatants harvested at 24 h post infection from mock-infected and *Burkholderia* spp. infected RAW264.7 macrophages were assayed for the production of **(E)** nitrite and **(F)** IFN-β. *P*-values between species were calculated by Mann–Whitney *U* test. ^∗∗∗^*p*-value < 0.001. **(G)** densitometric quantification of phosphorylated STAT1. Immunoblots in **Figures 2–4** were performed concurrently from the same samples and loading control, GAPDH. The immunoblot data is representative of three independent trials. Bacterial CFUs represent the average of two biological replicates. Nitrite and IFN-β levels were computed by averaging three biological replicates.

RPMA revealed iNOS expression was elevated 1 h post *Burkholderia* spp. infection, then reduced at 4 h and restored by 8 h (**Figure 2A**). Immunoblotting of independently prepared lysates samples revealed robust up-regulation of iNOS 8 h post *Bm* ATCC2334 and *Bm* 2002721278 infection. Furthermore, *Bp* E8 was a weaker inducer of iNOS expression when compared to both *Bm* strains (**Figures 2B,C**). Aminoguanidine (AG), an iNOS inhibitor, was utilized to correlate the importance of iNOS activity with clearance of intracellular *Burkholderia* spp. in RAW264.7 macrophages. The colony formation assay conducted 3 h post *Burkholderia* spp. infection suggested AG did not affect the uptake of bacteria. Treatment with AG augmented or restored replication of *Bm* strains in RAW264.7 macrophages 24 h post infection. Conversely, AG did not impact bacterial replication in *Bp* strains (**Figure 2D**). The efficacy of AG mediated inhibition of iNOS was further confirmed by quantifying nitrite production. AG inhibited *Bm* mediated nitrite production by approximately 80% whereas only basal levels of nitrite production was observed in *Bp* (**Figure 2E**). This is consistent with reported observations that iNOS expression was strongly induced when infected by *Bm* but not *Bp* (Utaisincharoen et al., 2001; Brett et al., 2008).

Infection of macrophages by Gram-negative bacteria induces the production of IFN-β (Utaisincharoen et al., 2003), which acts in autocrine or paracrine fashion and triggers subsequent STAT1 phosphorylation (Gao et al., 1998). Attenuation of iNOS gene expression in LPS stimulated, IFN-β deficient macrophages underscores the importance of IFN-β in modulating host innate immune responses (Thomas et al., 2006). To examine whether a relationship exists between IFN-β production and the induction of iNOS in the strains included in our study, culture supernatants were assayed for IFN-β by ELISA. When grouped, *Bm* infected RAW264.7 macrophages showed significant elevation of IFN-β production when compared to the *Bp* group (*Bp* E8 and *Bp* 576; *p*-value < 0.001) and *Bp* and *Bt* (*Bp* E8, *Bp* 576, *Bt* DW503) as a group (*p*-value < 0.00013). Furthermore, *Bt* was the least potent inducer among all the strains inspected in this study (**Figure 2F**). IFN-β mediated signal activation was further confirmed by immunoblotting for levels of STAT1 phosphorylation. Quantification of the phospho-STAT1 immunoblot shows increased activation of STAT1 in *Bm* strains versus *Bp*, which demonstrates distinct signaling dynamics between species (**Figures 2B,G**).

### *Burkholderia* spp. Induced NF-κB Mediated Responses

**FIGURE 3:**
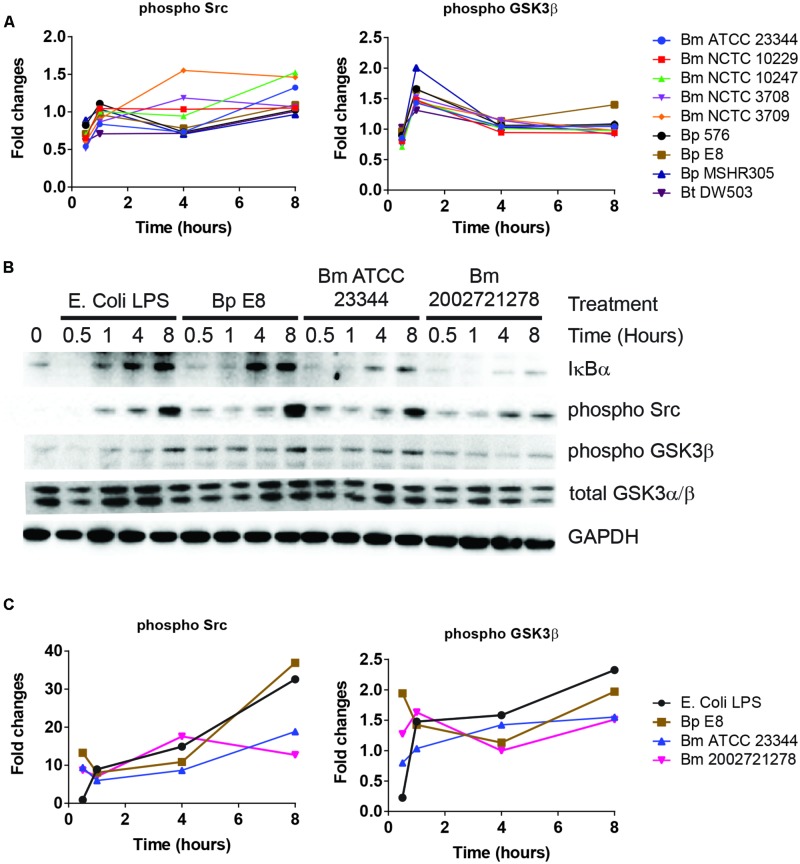
***Burkholderia* spp. induces NF-κB mediated responses. (A)** The phosphorylation state of indicated proteins acquired by RPMA. **(B)** Samples were prepared as described in **Figure 2B**. The expression or phosphorylation state of indicated proteins was evaluated by immunoblotting, followed by **(C)** densitometry quantification. Immunoblots in **Figures 2–4** were performed concurrently with the same sample and loading control, GAPDH. The immunoblot data is representative of three independent trials.

NF-κB pathway constitutes an important part of the host defense against pathogens (Akira et al., 2006). Several key regulators of NF-κB signaling pathway were also identified by RPMA. These include the phosphorylated forms of Src and GSK3β (Sasaki et al., 2005; Steinbrecher et al., 2005; Han et al., 2010). RPMA detected changes in Src and GSK3β phosphorylation (**Figure 3A**). The activities of these proteins were later characterized by immunoblotting. Phosphorylated Src, an integrin associated non-receptor tyrosine kinase, accumulated over time throughout the course of the study (**Figures 3B,C**). Furthermore, the level of phosphorylated GSK3β at serine 9 and 21, conferring the inactive form, increased over time in RAW264.7 macrophages treated with LPS, infected by *Bm* ATCC23344 and *Bp* E8. No apparent accumulation of phosphorylated GSK3β was observed in *Bm* 2002721278 infected RAW264.7 macrophages (**Figures 3B,C**).

Activation of NF-κB first requires phosphorylation of IκBα, which primes IκBα for ubiquitination and proteasome-mediated degradation. NF-κB subsequently translocates to the nucleus and activates a series of genes involved in the inflammatory response (Thanos and Maniatis, 1995). Although no dramatic changes in the expression level of total IκBα or phosphorylated IκBα were observed in the RPMA dataset, immunoblot analysis showed that cell lysates from LPS treated RAW264.7 macrophages were void of IκBα at 30 min post stimulation; however, it was quickly resynthesized by the 1 h time point. The expression level of IκBα remained intact at 4 and 8 h under LPS stimulation. After *Bp* and *Bm* infections, IκBα expression was absent at 30 and 60 min but was detected by 4 h, and maintained at 8 h. The avirulent *Bm* 2002721278 strain showed reduced expression of IκBα compared to the virulent strains (**Figure 3B**).

### Alternation of Intracellular Energy Level upon *Burkholderia* spp. Infection

**FIGURE 4:**
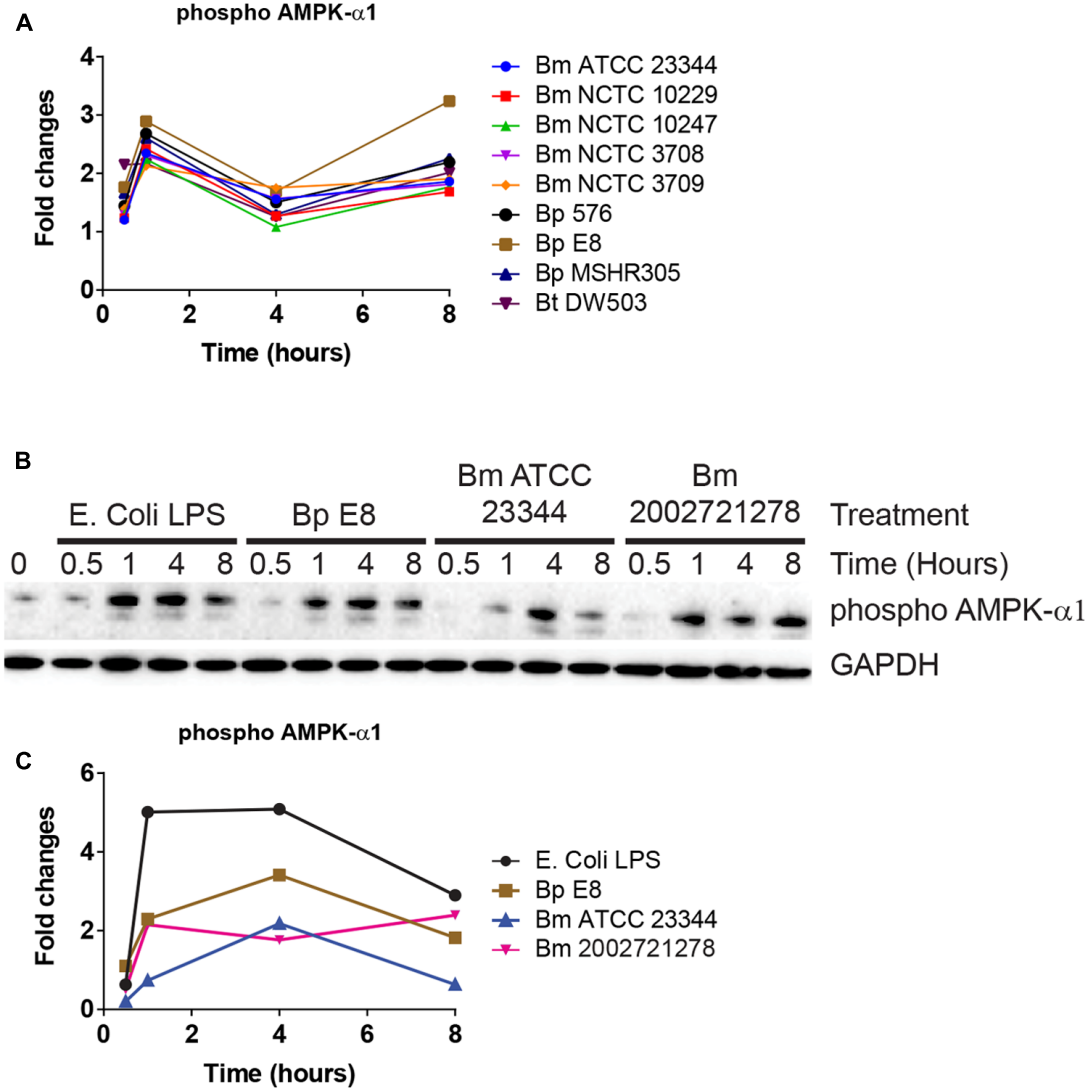
**AMPK-α1 is phosphorylated upon *Burkholderia* spp. infection. (A)** The phosphorylation state of AMPK-α1 acquired by RPMA. **(B)** Samples were prepared as described in **Figure 2B**. The phosphorylation state of AMPK-α1 was evaluated by immunoblotting, followed by **(C)** densitometry quantification. Immunoblots in **Figures 2–4** were performed concurrently with the same sample and loading control, GAPDH. The immunoblot data is representative of three independent trials.

AMPK-α1 is a serine/threonine protein kinase that has emerged as a master sensor of cellular energy balance in mammalian cells (Carling et al., 2011). However, post-translational modification of AMPK-α1 has not been implicated in the pathogenesis of *Burkholderia* spp. infections. The abundance of AMPK-α1 phosphorylation at an inhibitory site was altered in RPMA (**Figure 4A**). Immunoblot analyses confirmed increased expression of this phosphorylated form of AMPK-α1 after 1 h, which remained elevated throughout LPS stimulation or *Burkholderia* spp. infections (**Figures 4B,C**).

### Activation of MAPK Pathway in Response to *Burkholderia* spp. Infections

**FIGURE 5:**
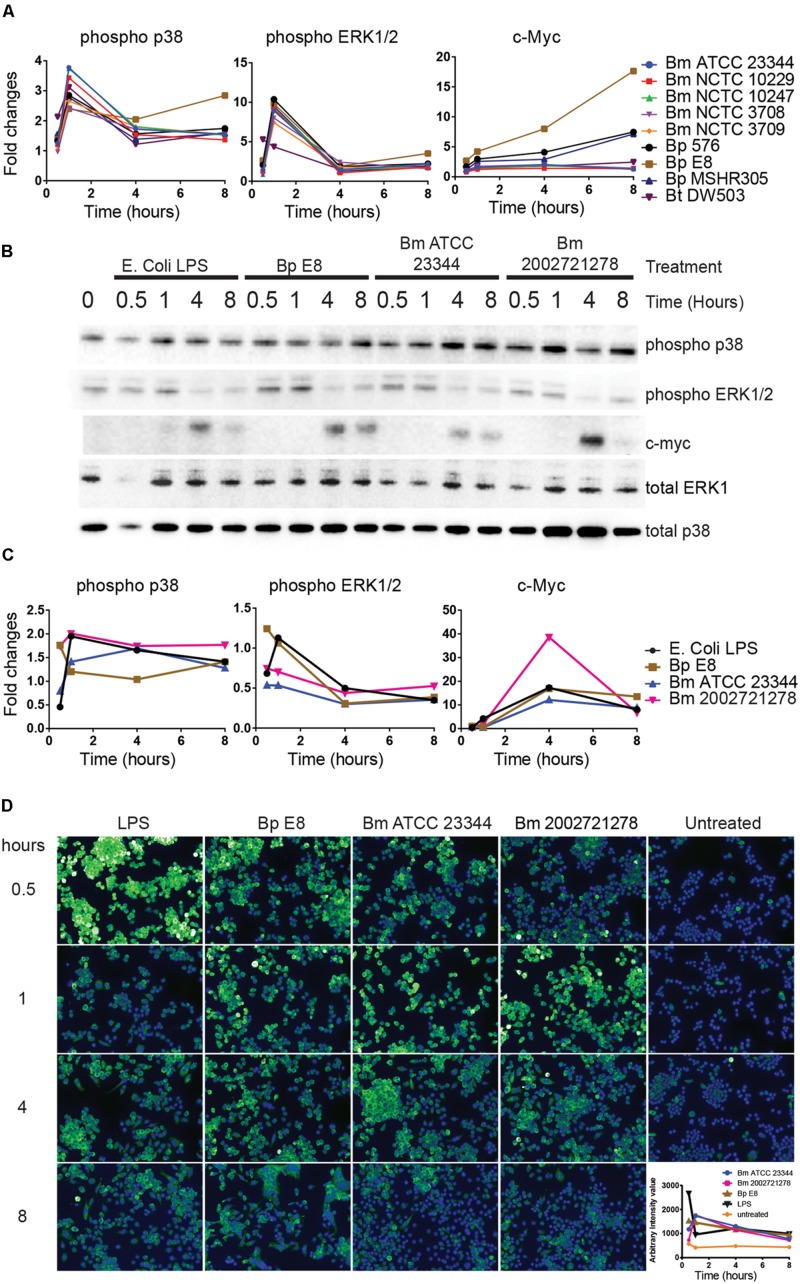
**Activation of MAPK pathway components upon *Burkholderia* spp. infection. (A)** The expression and phosphorylation states of indicated proteins acquired by RPMA. **(B)** Samples were prepared as described in **Figure 2B**. The expression and phosphorylation states of indicated proteins were evaluated by immunoblotting, followed by **(C)** densitometry quantification. **(D)** RAW264.7 macrophages were stimulated with lipopolysaccharide (LPS) or infected with *Burkholderia* spp. at indicated time points. Phosphorylation of ERK1/2 was visualized by indirect immuno-fluorescent staining. Expression of phosphorylated ERK1/2 was subsequently quantified using the Opera confocal system. Phosphorylated ERK1/2 is pseudocolored green and nuclear stain is colored blue. The immunoblot data is representative of three independent trials.

Reverse-phase protein microarray identified multiple components of MAPK pathway. These included c-Myc and phosphorylated forms of ASK1, p38, and ERK (**Figures 1** and **5A**). Immunoblot analyses revealed elevated expression of phosphorylated p38 over the time course of treatments (**Figures 5B,C**). Both immunoblotting and immuno-fluorescent assay showed an increase in phosphorylated ERK at 30 min and 1 h post treatment (**Figure 5D**). This expression pattern matched the RPMA result where the phosho-ERK1/2 signal peaked at 1 h. c-Myc, a downstream transcription factor of p38, was robustly up-regulated 4 h post LPS stimulation and *Burkholderia* spp. infection. c-Myc expression was sustained 8 h post *Bm* ATCC23344 and *Bp* E8 infection but was attenuated in macrophages infected with *Bm* 2002721278 or treated with LPS (**Figures 5B,C**).

## Discussion

### Identification of Gross Changes in Host Protein Expression and Phosphorylation During *Burkholderia* spp. Infection

In this study, we undertook quantitative analysis of phosphoprotein signaling induced by *Burkholderia* spp. in RAW264.7 macrophages using an RPMA platform. This approach allowed us to simultaneously analyze changes in phosphorylation states or total protein levels for 114 protein species, across nine *Burkholderia* isolates, at two MOI and four different time points. We did not observe any strain specific up/down regulation or activation of proteins queried at either MOI (**Supplementary Figure S1**). RPMA screening identified 25 candidate proteins whose expression or phosphorylation states were altered during *Burkholderia* spp. infections. The observed differences in the host signaling are not a result of defects in bacterial entry or replication within macrophages, as all the *Bp, Bm*, and *Bt* strains were able to efficiently replicate within macrophages (data not shown; Pilatz et al., 2006; Brett et al., 2008; Wand et al., 2011). Eight proteins were selected for further characterization by immunoblotting. Although both RPMA and immunoblotting approaches revealed changes in protein expression levels after *Burkholderia* spp. infection, the kinetic expression profiles of these proteins were slightly different. For example, phosphorylated AMPK-α1 signal obtained from RPMA peaked at 1 h post infection, attenuated by 4 h and restored by 8 h. On the contrary, immunoblot analyses showed a time dependent increase of phosphorylated AMPK-α1 with a peak at 4 h. The observed difference may be attributed to the existence of variations in the microenvironment of the immobilized cell extracts within and across arrays, such as possible spatial inconsistencies on the nitrocellulose surface, non-uniform reagent treatment, or printing inconsistencies (Anderson et al., 2009). Furthermore, fundamental differences exist between the RPMA and immunoblot sample preparation and experimental methods. For example, the fluorophore used in the RPMA platform is highly sensitive with a broad dynamic range compared to the chemiluminescence detection system, resulting in different relative signal outputs. These variations may contribute to the disparity we observe in peaks of expression between the two assays. Despite the variation between the observed protein expression kinetics, our goal to identify critical host factors is not undermined.

The signaling pathways altered in *Burkholderia* spp. infected RAW264.7 macrophages include AMPK-α1, regulators of NF-κB signaling pathway (IκBα, GSK3β, Src, STAT1) and MAPK (p38, ERK1/2, c-Myc). Notably, RPMA platform study by Molero et al. (2009) revealed both MAPK and GSK-3β activities are exploited by *Salmonella enterica* Serovar Typhimurium (*S*. Typhimurium) encoded type III secretion systems (T3SS) during the internalization process. Internalization of *S*. Typhimurium is dependent on the function of Cdc42- and Rac1-activating factors SopE/E2, which promotes actin rearrangement. A third effector protein, SigD, which also contributes to bacterial internalization, triggered Akt activation which subsequently led to GSK-3β inhibition by phosphorylation on serine 9. Cells pretreated with a p38 inhibitor showed significantly reduced *Bp* entry; however, the exact component of the T3SS that mediates this process has yet to be identified (Hii et al., 2008). The ability of *Burkholderia* spp. to alter phosphorylation states of several MAPK family members and GSK-3β (Serine 9) suggests both MAPK and GSK-3β are within a conserved axis exploited during the *Burkholderia* pathogenesis.

Manipulation of host kinase signaling by bacterial effectors is a major mechanism in the pathogenesis of infection (Krachler et al., 2011). It was reported that *Bp* T3SS contribute to activation of host proteins such as NF-κB and JNK in a TLR-independent manner although the specific bacterial effectors have not been identified (Hii et al., 2008). Presently, there is limited information linking the specific bacterial effectors to host pathways. While it is beyond the scope of this study, elucidating the bacterial-host networks will be critical for identification of host proteins that could be targeted for development of host-based therapeutics.

### Induction of iNOS in Response to *Burkholderia* spp. Infection

Induction of iNOS upon bacterial infection of macrophages is a host response reported to enhance antimicrobial activity through activation of the TLR4 pathway as well as IFN signaling (Jacobs and Ignarro, 2001; Utaisincharoen et al., 2004; Brett et al., 2008). iNOS catalyzes production of nitric oxide (NO), which is oxidized to reactive nitrogen oxide species that play an important role in the clearance of intracellular bacteria (Coleman, 2001). RAW264.7 cells infected with *Bm* ATCC23344 and *Bm* 2002721278 showed reduced levels of total IκBα in comparison with *Bp* E8, suggesting elevated NF-κB activity upon *Bm* infection. Moreover, production of IFN-β was elevated in *Bm* infected RAW264.7 cells compared to *Bp* strains. This suggests upregulation of iNOS protein expression observed in *Bm* strains may thus be a result of the activity orchestrated by NF-κB and IFN-β. In fact, multiple transcription factors such as NF-κB, STAT1, and interferon regulatory factor (IRF) are demonstrated to regulate the iNOS promoter (Taylor and Geller, 2000). Notably, our results demonstrate that differential expression of iNOS in *Bm* vs. *Bp* strains plays a role in bacterial clearance as evidenced by bacterial survival in the presence of AG.

### Potential Targets for Anti-Inflammatory Therapeutics in *Burkholderia* spp. Infection

Balancing the inflammatory network may direct the host responses toward an anti-inflammatory state and may represent a more effective means of treatment for postsymptomatic infections (D’Elia et al., 2013). Phosphorylation of AMPK-α1, Src, and GSK3β upon *Burkholderia* spp. infection suggested their involvement in modulating host inflammatory response. First, suppression of AMPK-α1 activation via Serine 485 phosphorylation was observed in *Burkholderia* spp. infected cells. This suggested that a pro-inflammatory mechanism was quickly mounted in response to the infection. Potentiating AMPK-α1 activation may mitigate the inflammatory response through promoting oxidative metabolism, which is connected to an anti-inflammatory state, rather than the glycolytic state associated with inflammation. Secondly, the tyrosine kinase Src mediated integrin signaling pathway not only modulates leukocyte adhesion and migration but also negatively regulates TLR4 activation (Han et al., 2010). In this context, activation of Src implicates initiation of a TLR4 mediated negative feedback mechanism. Attenuating activation of NF-κB through potentiating Src mediated integrin signal cascade may thus serve as a therapeutic route for *Burkholderia* spp. infection. Thirdly, reduced transcriptional activity of NF-κB was associated with GSK3β inhibition and this resulted in attenuation of TLR mediated inflammatory cytokine production (Martin et al., 2005). *Bp* infected mice pretreated with GSK3β inhibitor showed reduced levels of pro-inflammatory cytokines (TNF-α, IFN-γ, IL-1β) and elevated levels of anti-inflammatory cytokines (IL-10 and IL1Ra). Strikingly, *Bp* infected mice showed improved survival when treated with the GSK3β inhibitor (Tay et al., 2012). This selective action of GSK3β on NF-κB-induced gene expression will likely facilitate the therapeutic anti-inflammatory uses of GSK3β inhibitors.

### *Burkholderia* spp. Host Signal Transduction Network

**FIGURE 6:**
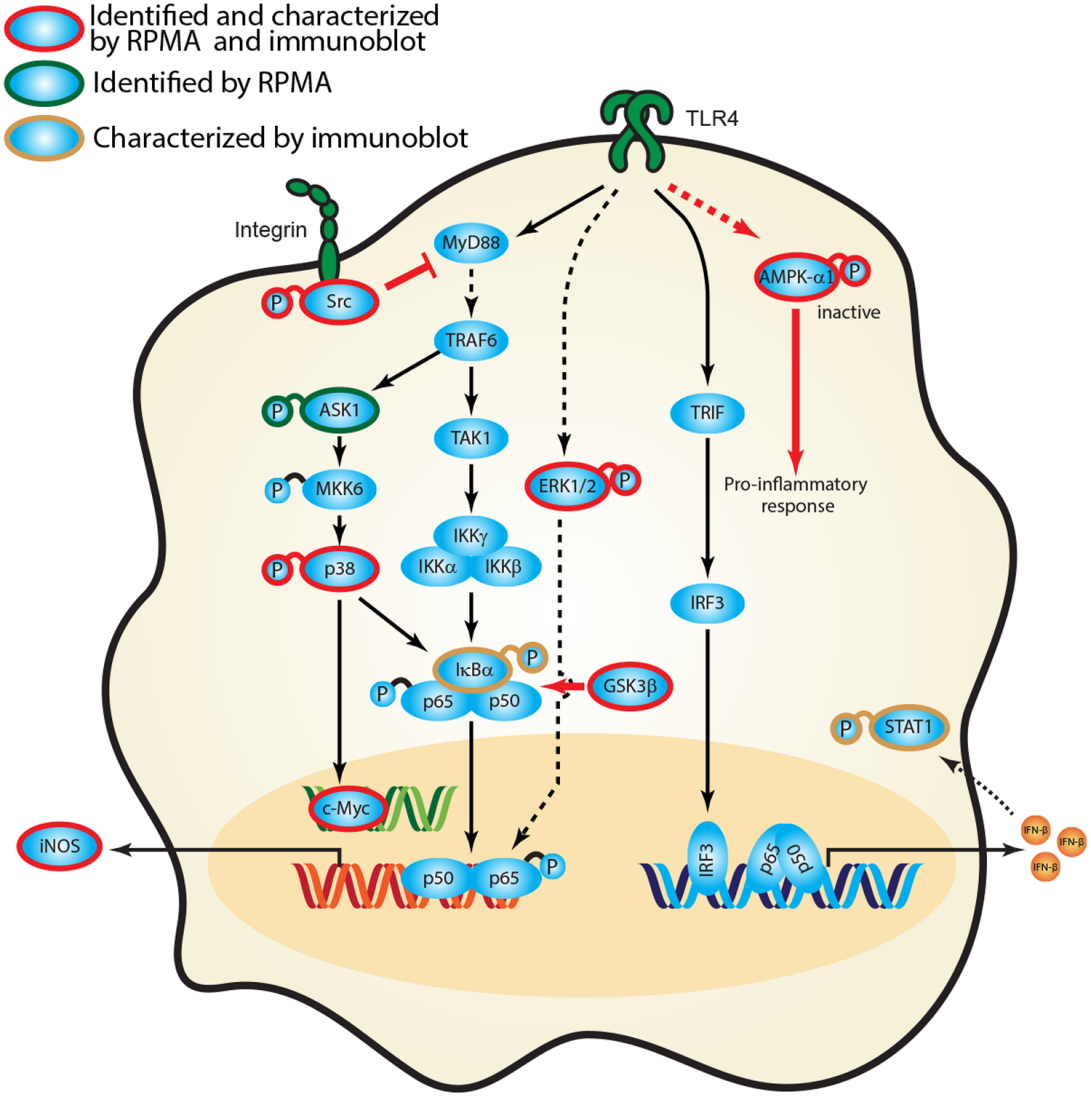
**Activation of signaling cascades in response to *Burkholderia* spp. infection.** A signaling network was constructed based on current knowledge of signal transduction pathways and our survey of host protein expression and phosphorylation profiles in response to *Burkholderia* spp. infection. Proteins identified in the RPMA screen (green circle) and/or characterized by immunoblotting are designated by color coding (gold circle, confirmed by immunoblot; red, confirmed by RPMA and immunoblot). Red bold arrow represents potential therapeutic routes.

Empirical studies have characterized the function of numerous host proteins in the context of *Burkholderia* spp. infection; however, they have not been queried systematically against multiple *Burkholderia* spp. Our high throughput RPMA approach successfully identified several previously published protein targets as well as new, unreported host proteins that were altered in response to *Burkholderia* spp. infection. To the best of our knowledge, activation of Src, inactivation of AMPK-α1 or expression of c-Myc has not been implicated as altered in *Burkholderia* spp. infection. We have constructed a representative network that encompasses critical signaling axes based on current framework of signal transduction pathways and our survey of host protein expression and phosphorylation profiles. Canonical pathways downstream of TLR4 include several host proteins from our studies, namely IκBα, active Src, ERK1/2, p38, and inactive AMPK-α1. The aforementioned kinases ultimately activate transcription factors (e.g., c-Myc, STAT1, and NF-κB) which serve to regulate gene expression (e.g., iNOS) during *Burkholderia* spp. infection (**Figure 6**). Based on our results, induction of phosphorylated forms of AMPK-α1, GSK3β, and Src in the context of canonical pathways suggest that they play a role in regulating the inflammatory response of *Burkholderia* spp. infections. Inhibiting AMPK-α1 (by phosphorylating Serine 485) triggers a pro-inflammatory mechanism whereas inhibiting GSK3β (by phosphorylating Serine 9) may result in suboptimal NF-κB mediate inflammatory response. In addition, activating integrin associated Src kinase may negatively regulate MyD88 dependent NF-κB activation (**Figure 6**). Importantly, perturbing the nodes in these pathways by antisense technology and bioactive small molecules will serve to validate these host factors in the pathogenesis of *Burkholderia* spp. infections.

## Conflict of Interest Statement

The Associate Editor Kurt John Langenbach declares that, despite being affiliated to the same institution as author Ramin M. Hakami, the review process was handled objectively and no conflict of interest exists. The authors declare that the research was conducted in the absence of any commercial or financial relationships that could be construed as a potential conflict of interest.

## Supplementary Material

FIGURE S1 **Heat map of fold changes over mock treated samples.** RAW264.7 macrophages were infected with indicated Burkholderia spp. for 0.5, 1, 4, 8 h. Lysates were harvested and subjected to RPMA methodology. The expression patterns and phosphorylation states of indicated proteins after infection with an MOI of 10 **(A)** or MOI of 1 **(B)** are depicted.

TABLE S1 **Antibodies used in RPMA experiment.** Explanations of column headings are: Name of antibody: name of the proteins that antibodies recognize; Vendor: the vendor of the antibodies; Catalog #: the catalog number associated with the vendor; Molecular Weight: the size of the protein in kilo-Dalton; Host: the source of the antibodies; Dilution: dilutions used in the PRMA.
