# Supplementary figures and images for: A reverse-phase protein microarray-based screen identifies host signaling dynamics upon Burkholderia spp. infection
Source: Front Microbiol. 2015 Jul 27;6:683. doi: 10.3389/fmicb.2015.00683 (PMC4515560; doi:10.3389/fmicb.2015.00683)

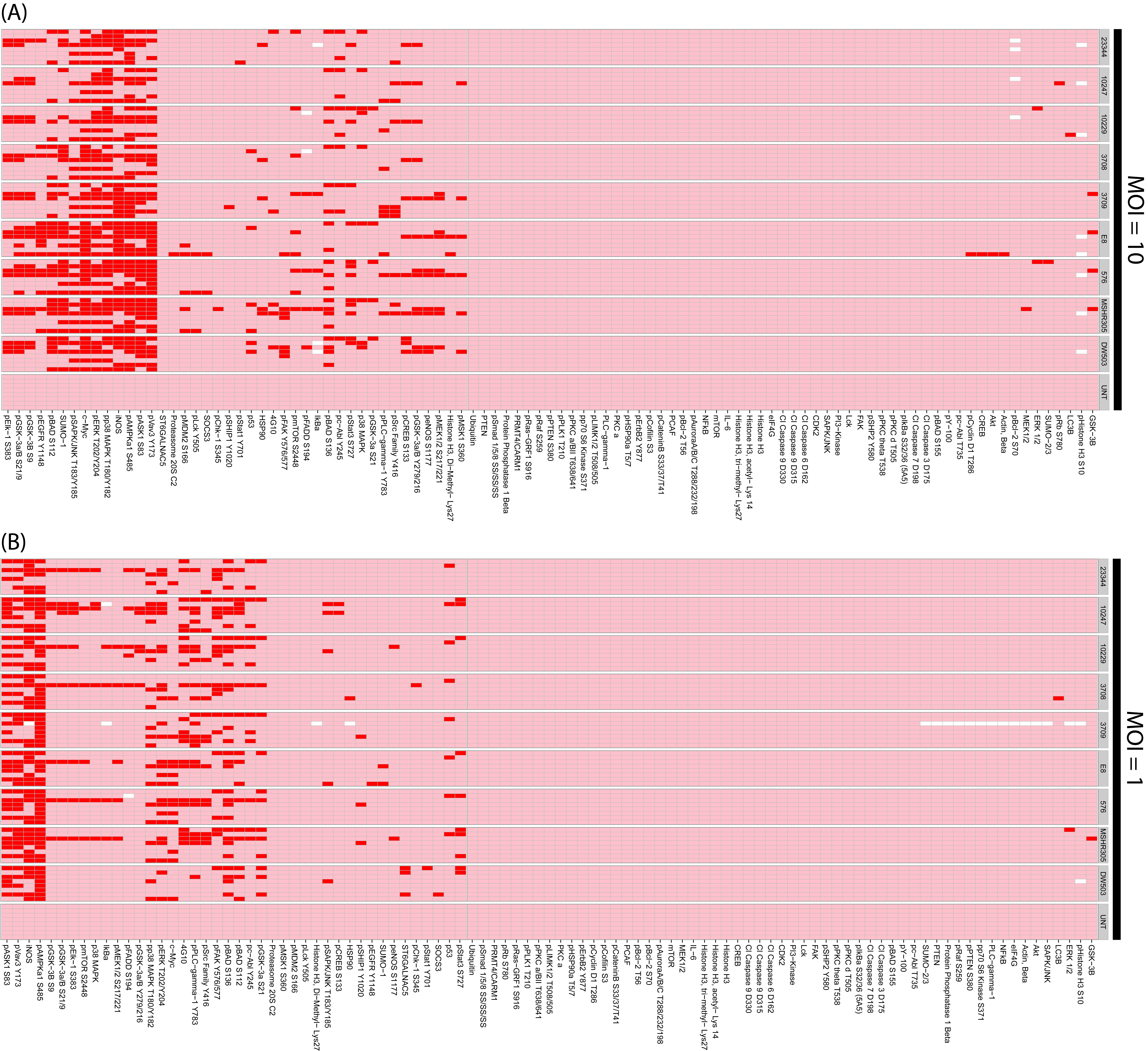

Supplement: FIGURE S1 — Heat map of fold changes over mock treated samples. RAW264.7 macrophages were infected with indicated Burkholderia spp. for 0.5, 1, 4, 8 h. Lysates were harvested and subjected to RPMA methodology. The expression patterns and phosphorylation states of indicated proteins after infection with an MOI of 10 (A) or MOI of 1 (B) are depicted. [file Image_1.JPEG]
